# Fracture Resistance and Surface Roughness of Zirconia Crowns Veneered by Layering, Press-on, and CAD-on Methods 

**DOI:** 10.30476/dentjods.2025.105198.2579

**Published:** 2026-09-01

**Authors:** Mina Mohaghegh, Dorsa Pourzarabi, Masumeh Taghva, Reza Derafshi

**Affiliations:** 1 Dept. of Prosthodontics, Biomaterials Research Center, School of Dentistry, Shiraz University of Medical Sciences, Shiraz, Iran.; 2 Student, School of Dentistry, Shiraz University of Medical Sciences, Shiraz, Iran.; 3 Dept. of Prosthodontics, School of Dentistry, Shiraz University of Medical Sciences, Shiraz, Iran.

**Keywords:** CAD-on, Fracture resistance, Layering, Press-on, Surface roughness, Zirconia

## Abstract

**Background:**

Various strategies are used for ceramic veneering on zirconia copings. Veneering techniques may influence the fracture resistance and surface roughness of zirconia crowns and controversial results are present in literature concerning these techniques.

**Purpose:**

The aim of the present study was to investigate the effects of layering, press-on, and CAD-on techniques on the fracture resistance and surface roughness of zirconia crowns after aging.

**Materials and Method:**

In this *in vitro* study, 30 brass dies were milled and scanned. Coping was designed for all of them, milled from a zirconia block, and sintered. Porcelain veneering was done through layering, press-on, and CAD-on methods (n=10 per group), and aged in autoclave. Surface roughness was measured before and after aging. Fracture resistance was tested, and the failure pattern was checked by scanning electron microscopy (200-500×). The independent t-test and post-hoc test were used to compare the fracture resistance among the three groups. The surface roughness before and after aging was investigated using one-way ANOVA and ANCOVA. The data on failure patterns were analyzed using the chi-square test.

**Results:**

The mean fracture resistance in the CAD-on group (4442.19±1580.62 N) was higher than in layering (3225.63±1266.08 N) and press-on groups (2652.42±1320.02 N). The press-on group exhibited the highest surface roughness both before (2.97±0.26μm) and after aging (3.33±0.83μm). The most common failure patterns were adhesive pattern in the CAD-on group and cohesive pattern in layering and press-on groups.

**Conclusion:**

The three veneering techniques had clinically acceptable fracture resistance and surface roughness. The highest fracture resistance was found in the CAD-on group, followed by the layering and press-on groups. The lowest surface roughness was observed in the layering group, followed by CAD-on and press-on groups. Techniques with higher fracture resistance can be considered in patients with high force factors such as bruxism, clenching, and long span bridges.

## Introduction

Zirconia is one of the most recently introduced ceramic materials, which has become one of the most popular dental ceramics due to its ideal mechanical properties and ideal biocompatibility [ 1 - 3 ].

Zirconia crowns are suggested as a suitable option for posterior ceramic restorations. Despite the excellent strength of the zirconia coping, chipping of the veneering ceramic has been reported in 74 to 100 percent of cases [ 4 - 5 ]. This defect is attributed to various factors such as the sintering process of zirconia, structural defects, grinding damage during laboratory processes and aging of zirconia [ 6 - 7 ]. 

The opaque hue of zirconium oxide ceramics prevents perfect staining and distorts the esthetic aspect. Therefore, they should be veneered to improve their esthetic outcomes [ 8 ]. Various strategies are used for ceramic veneering on zirconia copings. In the conventional layering technique, ceramic powder and liquid are mixed and placed on the sintered zirconia coping, which is slightly larger. In the sintering process, the porcelain shrinks to the desired size [ 9 ]. However, this technique can be subjected to operator-related errors [ 4 ]. In the press-on technique, the desired contour is formed by wax on the sintered zirconia coping and then invested with pressable ceramics [ 9 ]. Alternatively, computer-aided design/computer-aided manufacturing (CAD/ CAM) technology can be used to form the wax or resin pattern of the veneer, invest with pressable ceramics and then be connected to the coping [ 10 ]. 

Currently in CAD-on technique, CAD/CAM technology is suggested for fabrication of both zirconia coping and overlaying veneer [ 4 ]. In this technique, both the zirconia coping and the veneer layers are designed in CAD software. Zirconia copings and lithium disilicate veneers are machined and sintered to a predetermined shape in a CAM process. Finally, they are attached by a low-fusing glass-ceramic. Another sintering cycle is used simultaneously for the crystallization of lithium-disilicate and the fusion process. The CAD-on method is less time-consuming and less susceptible to human performance inconsistencies [ 10 ]. It aids in the fabrication of homogeneous zirconia copings and veneers without imperfections or porosities that can lead to clinical failures [ 11 ]. 

The veneering technique may affect the fracture resistance of zirconia crowns. However, controversies exist in literature according to fracture resistance of various methods [ 12 - 14 ].

The other determining feature of zirconia crowns is surface roughness. Increased surface roughness leads to some problems such as the wear of the opposing dentition [ 7 , 15 ] and the additional plaque accumulation [ 16 - 17 ]. The physical factors related to the abrasiveness of the dental materials include hardness, frictional resistance, porosity, crystal size, and fracture toughness [ 12 , 18 ]. Zirconia crowns veneered with various methods and materials differ in surface roughness [ 16 ]. Therefore, it is essential to know the roughness value of crowns fabricated through different materials and methods.

Another limiting factor is aging of yttrium-stabilized tetragonal zirconia ceramic due to its potential sensitivity to low-temperature degradation) or aging [ 19 - 20 ]. This phenomenon is caused by the transformation of tetragonal phase to monoclinic phase in the presence of water [ 21 ], which may degrade the surface, weaken the mechanical properties, and leads to treatment failure [ 22 ].

Factors such as stabilizer type, grain size, and residual stress can affect aging. There is limited data on the effects of aging of zirconia restorations used for oral rehabilitation. The aim of the present study was to evaluate the effects of different veneering techniques on the fracture resistance and surface roughness of zirconia crowns after aging.

The null hypothesis is that the various techniques used for veneering zirconia crowns (layering, press-on, CAD-on) do not produce significant differences in fracture resistance and surface roughness.

## Materials and Method

**Figure 1:**
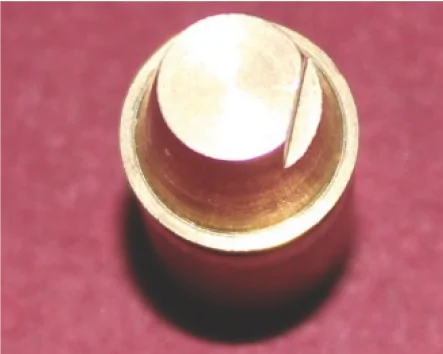
Brass metallic die

The Institutional Review Board (IRB) approval was obtained for this experimental *in vitro* study (IR.SUMS. REC.1398.376).In this study, 30 brass dies were milled with a CNC machine (CNC350; Arix Co. Tainan Hesin Taiwan) in standard size for all-ceramic crowns with 6˚occlusal convergence, 1 mm wide shoulder finish line, and axial anti-rotational design to ensure identical seating of the copings on the die (Figure 1) [ 23 ]. 

**Figure 2:**
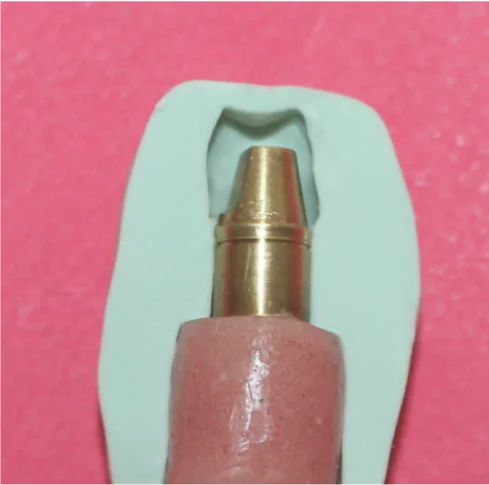
The putty index to ensure that the dimensions of all specimens are identical

The metal dies were coated with scanning spray, and scanned with a 3D-Laser scanner (3ShapeD18; 3Shape, Copenhagen, Denmark). Data were transferred to CAD software (3Shape CAD Design Software; 3Shape, Copenhagen, Denmark), and the copings were designed with a uniform thickness of 0.5mm around and 30μm spacer, 1mm from margin. The copings were designed 20% larger to compensate for polymerization shrinkage [ 10 ]. The zirconia copings were fabricated in a milling machine (Inlab MC XL, Dentsply Sirona, USA) from pre-sintered zirconia blocks (IPS e.maxZir CAD, Ivoclar Vivadent, Schaan, Liechtenstein), and sintered in the synthesis furnace at 1580˚C (Programat S1, Ivoclar Vivadent, Germany). The specimens were divided into three groups (n=10) and veneered with porcelain by layering, press-on, and CAD-on methods. For layering method, porcelain powder was mixed with the specific liquid to make the veneers through layering method. The obtained paste was applied over the copings with a brush in several steps. This technique consisted of four sintering stages: 1) the liner (IPS e.max, Zirliner; Ivoclar Vivadent AG, Germany), which was fired in a 960˚C furnace, 2) the first layer of dentin and enamel, which was a nano-fluoro-apatite-glass-ceramic (IPS e.max Ceram; Ivoclar Vivadent AG, Schaan, Liechtenstein) processed at 750˚C, 3) the second layer of dentin and enamel, and 4) the glaze [ 24 ]. A single skilled technician did the veneering of all the copings. The thickness of the veneering material was 0.7mm at the margin and a maximum of 1.5mm on the occlusal surface. The restorations were formed slightly larger to compensate for polymerization shrinkage. A putty index was prepared and used to ensure that the dimensions were identical for all specimens (Figure 2). 

**Figure 3:**
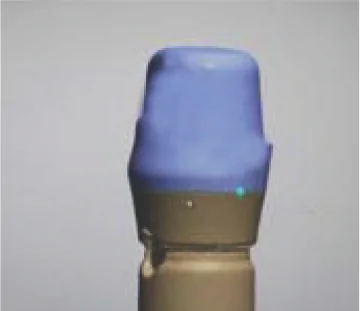
The design of veneer for press-on method with a thickness of 0.7 mm at the margins and a maximum thickness of 1.5mm at occlusal surfaces

In order to create veneers using the press-on method, the external surface of a crown from the layering group, and the external surface of zirconia coping were scanned. The veneering material was designed (3Shape's CAD Design software; 3Shape, Copenhagen, Denmark) with a thickness of 0.7mm at the margins and a maximum thickness of 1.5mm at occlusal surfaces (Figure 3). Resin replicas of the veneers were milled out of casting acrylate polymer blocks (IPS AcrylCAD; Ivoclar Vivadent) and attached to the zirconia copings with a casting wax. The previously mentioned putty index was used to ensure identical dimensions in all specimens. The sprue was attached to the complex, placed in the ring, invested, and allowed to set for nearly 40 minutes. The rings were heated at 900˚C for 60 minutes to burn out the wax and resin. Then, the ceramic (IPS e.max-ZirPress, Ivoclar Vivadent AG, Schaan, Liechtenstein) was pressed into the mold and sintered at 910˚C in a furnace (Programat EP 5000; Ivoclar Vivadent, Germany).The muffles were allowed to dry at room temperature. The investment material was removed by a diamond bur and sandblasting with 50µm particles. The sprue was separated; the crowns were stored in acidic solution (IPS e.max press Invex Liquid, Ivoclar Vivadent AG, Schaan, Liechtenstein) in the ultrasonic unit for 5 minutes, rinsed with water and dried. The ceramic veneers were contoured and finished. They were steam-cleaned and glazed by applying the glaze paste (IPS e.max Ceram Glaze Paste, Ivoclar Vivadent AG, Schaan, Liechtenstein) on the crown surface at 750˚C [ 10 , 25 ]. 

**Figure 4:**
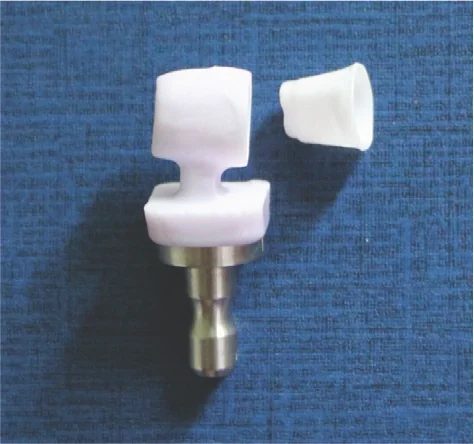
The veneer and coping parts in CAD-on technique

The third technique was CAD-on, which blocks of pre-crystallized lithium disilicate glass-ceramic (IPS e.max CAD, Ivoclar Vivadent, Schaan, Liechtenstein) were used together with CAD/CAM technology (inLab MC XL, Dentsply Sirona, USA). Identical veneering dimensions in the layering and press-on groups were maintained by using the veneer already designed for the press-on group (Figure 4). The inner surface of the veneer and the outer surface of the fabricated coping were covered with low-viscosity adhesive (IPS e.max CAD Crystall, Connect 3 Ivoclar Vivadent, Schaan, Liechtenstein), and excess material was removed with a brush. 

The veneer and coping were carefully pressed together. Crystallization of the machined pre-crystalline ceramic veneer required sinter bonding at 850˚C in a ceramic sintering furnace. Sintering caused adhesion of the veneer to the coping. The glaze (IPS e.max Ceram Glaze & IPS e.max CAD Crystall/Glaze) was applied at 725˚C. The previously mentioned putty index was used to ensure identical dimensions.

The surface roughness of the prepared specimens was measured using a profilometer (TESA Rugosurf 20, Switzerland). The average roughness parameter (Ra) was measured (µm) and cut-off was set at 0.8. In order to simulate the effects of low temperature degradation, the specimens were aged in an autoclave (Keyhan, MFG, Tehran, Iran) at 134˚C and 2 bar pressure for 3 hours, which was equal to 10 years of clinical use of the restorations at 37˚C in oral condition [ 21 - 22 ]. Surface roughness was measured again after aging. 

**Figure 5:**
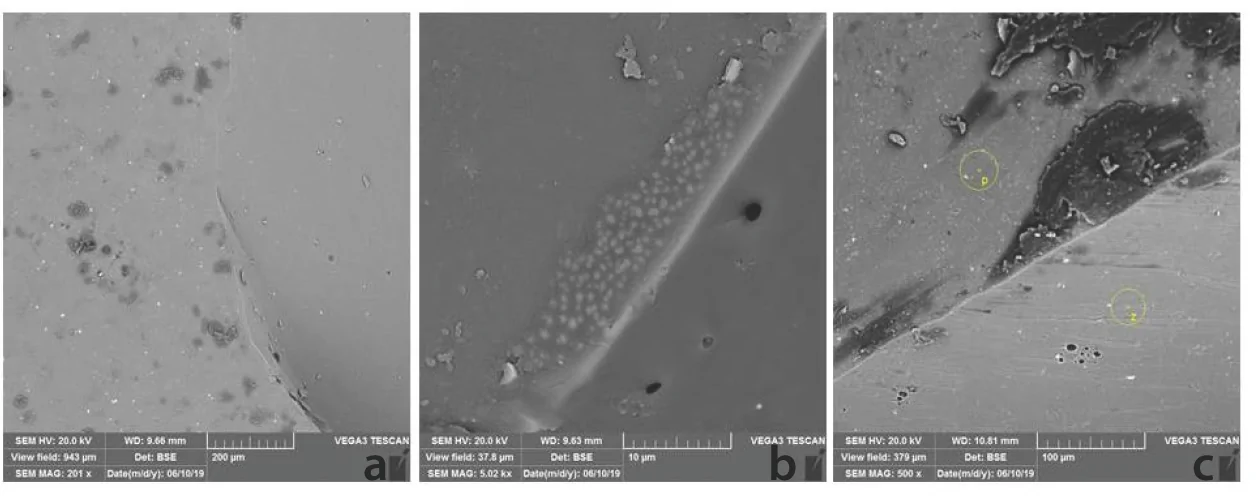
Electron microscope scans (SEM, TESCAN Vega3, Czech Republic) at 200× to 500× magnification of the fractured samples. **a:** Adhesive failure pattern, **b:** Cohesive failure pattern, **c:** Mixed failure pattern

To measure the fracture resistance, specimens were cemented on metal die with glass-ionomer cement (GC Gold Label Glass Ionomer, Luting & Lining Cement, Japan). The cement was mixed according to the manufacturer's instructions, placed in the crown with an applicator, fixed on the corresponding brass die, and the excess cement was removed. Perfect seating was ensured after the complex was loaded with 5kg for 10minutes [ 23 ]. To assess the fracture resistance using the Instron testing machine (Zwick/Roell, Z020, Germany), a vertical force was applied to the occlusal central fossa at a crosshead speed of 0.5mm/min until fracture occurred. To assess the failure pattern (adhesive/ cohesive), specimens were cleaned with alcohol and then examined with a scanning electron microscope (SEM, TESCAN Vega3, Czech Republic) at 200× to 500× magnification (Figure 5). An adhesive failure pattern was present when the failure occurred at the interface between the coping and veneer, exposing the zirconia coping; a cohesive failure pattern referred to the failure within the veneer, leaving a layer of the veneering ceramic on the zirconia coping. Finally, a mixed failure pattern was considered as a combination of adhesive and cohesive failure. 

### Statistical analysis

Data were statistically analyzed using SPSS software (version 22, SPSS Inc., Chicago, IL, USA). The fracture resistance and surface roughness before and after aging were reported descriptively as mean and standard deviation (Mean±SD). Due to the normality of the fracture resistance data, they were compared among the groups via independent t-test and post-hoc test. The normality test was performed for the surface roughness data and showed normal distribution of the results. One-way ANOVA and Tukey post hoc test were used to compare the three groups in terms of the surface roughness before aging. ANCOVA was used to compare the three groups in terms of the surface roughness after aging. In addition, a paired-sample t-test was used to compare surface roughness in each group before and after aging. Data on failure patterns were analyzed data was analyzed using the chi-square test. The 5% significance level was used for all statistical tests.

## Results

The highest fracture resistance value was recorded in the CAD-on group (4442.19±1580.62 N), followed by the layering group (3225.63±1266.08 N), and press-on group (2652.42±1320.02 N). The difference between the three veneering methods (*p*= 0.049) (Table 1) was statistically significant. Pairwise comparison of the study groups by post-hoc test revealed a significant difference between the press-on and CAD-on groups (*p*= 0.016), whereas there was no significant difference between layering and press-on (*p*= 0.437) and layering and CAD-on groups (*p*= 0.104) (Table 2).

**Table 1:** Comparison of the mean±standard deviation of fracture resistance values among the study groups

Groups	Fracture resistance (N)
L	3225.63±1266
P	2652.42±1320
C	4442.19±1580
*p* Value	0.049

**Table 2:** Pairwise comparison of the fracture resistance values among the study groups (Post Hoc Test)

Groups	Sig	Adj. Sig
L&P	0.437	1.000
P&C	0.016*	0.049*
L&C	0.104	0.312

L: layering, P: Press-on, C: CAD-on. * means significant difference between groups

**Figure 6:**
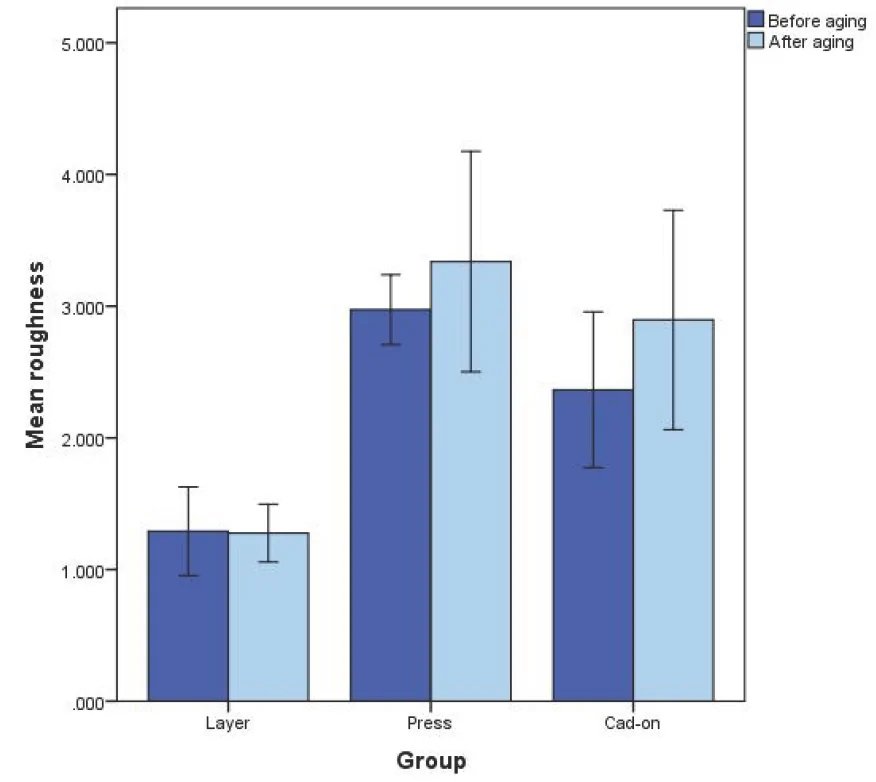
Mean surface roughness value (Ra) and 95% confidence interval among the three groups before and after aging

The mean surface roughness values of the three groups were significantly different before (*p*< 0.001) and after aging (*p*< 0.046). The pair-wise comparison showed that the mean surface roughness before aging was significantly lower in the layering group than in the press-on group (*p*= 0.000) and the CAD-on group (*p*= 0.000). It was also significantly lower in the CAD-on group than in the press-on group (*p*= 0.02) (Tables 3-4). The surface roughness after aging was significantly lower in the veneering group than in the press-on group (*p*= 0.02) and the CAD-on group (*p*= 0.01). However, there was no significant difference between the press-on and CAD-on groups (*p*= 0.45) (Tables 3-4, Figure 6). 

**Table 3:** Comparison of the study groups regarding the me-an ±SD values of surface roughness before and after aging

Groups	Surface roughness		*p* Value***
	Before aging	After aging	
L	1.29 ±0.33	1.27± 0.21	0.9
P	2.97± 0.26	3.33 ±0.83	0.1
C	2.36± 0.59	2.89± 0.83	0.2
*p* Value	<0.001*	0.046**	

L: layering, P: Press-on, C: CAD-on

* One-way ANOVA. (Tukey post-hoc test)

** ANCOVA. (LSD test)

*** Comparison of surface roughness values in each group before and after aging. (Paired T-test)

**Table 4:** Pairwise comparison of surface roughness of study groups before and after aging

Surface roughness	Groups		*p* Value
Before aging	L	P	.000
		C	.000
	P	L	.000
		C	.023
	C	L	.000
		P	.023
After aging	L	P	.025
		C	.015
	P	L	.025
		C	.457
	C	L	.015
		P	.457

L: layering; P: Press-on; C: CAD-on

The failure pattern through the test groups is displayed in Table 5. Based on fracture patterns, statistical analysis revealed no significant difference between the three veneering methods (*p*> 0.05).

**Table 5:** Frequency of failure patterns indifferent veneering methods after fracture resistance test

Failure pattern	L	P	C
Adhesive	1	2	6
Cohesive	7	6	1
Mixed	2	2	3
p Value*	0.199	0.223	0.199

Adhesive: the failure occurred at the coping and veneer interface, exposing the zirconia coping; cohesive: the failures occurred within the veneer, identified with a layer of veneering ceramic remaining on the zirconia coping. Mixed: a combination of adhesive and cohesive failure. L: layering; P: Press-on; C: CAD-on; * Chi-square test

## Discussion

The findings of this *in vitro* study showed that each of the layering, press-on and CAD-on techniques would result in zirconia crowns with significantly different fracture resistances. Techniques with higher fracture resistance can be considered in patients with high force factors such as bruxism, clenching and long span bridges.

Crowns fabricated with the CAD-on technique had significantly higher fracture strength than the press-on and layering techniques, as reported in several other studies [ 11 , 22 , 26 ]. CAD-on is a digital method, whose digital processing design makes it highly accurate and fracture resistant. This method was developed to reduce porosity and increase the fracture resistance of zirconia restorations. It is cost-effective since both the coping and the veneer are fabricated by the device, with the only intervention being the application of fusing material between the zirconia coping and the lithium disilicate veneer and subsequent firing [ 26 ]. 

Although there was no statistically significant difference in the fracture resistance of CAD-on and layered groups, the lower fracture resistance values of the layering group can be explained by more internal stresses at the veneer interface due to the coefficient of thermal expansion discrepancies. These internal stresses may contribute to the lower fracture resistance of the hand-layered zirconia crowns [ 27 ]. 

In the present study, fracture resistance values were lower in the press-on group than in the CAD-on and layering groups. This result agrees with the results of Ezzat *et al*. [ 28 ], who found higher fracture resistance in the layering technique than in the press-on technique. This result may be related to the fact that the entire veneer is fired over the coping in a single step, which may result in residual stresses remaining in the veneer and in the bonding area.

Beuer *et al*. [ 11 ] compared three veneering methods and found no difference between the fracture strength of single crowns fabricated by either pressing or manual veneering method. However, the CAD/CAM method, which used lithium disilicate blocks, showed significantly higher fracture strength than the other two other methods. Similar to the present study, Sim *et al*. [ 29 ] compared three techniques in the LAVA system and found the highest fracture strength in the digital method (CAD /CAM). Chaar *et al*. [ 12 ] compared the fracture resistance of 3-unit fixed zirconia restorations and found that the combination of CAD/CAM and press-on techniques had a higher fracture resistance after artificial aging.

Hung *et al*. [ 26 ] found that both the press-on and CAD/CAM techniques reduced fracture rates and exhibited higher fracture resistance than the layering technique.

In previous studies, wide ranges of fracture resistance have been reported (346-6263N), which can be explained due to different loading protocols, variable abutment materials, and existing luting cement. [ 28 ] The fracture resistance values in the current study were within the range reported by previous studies. Similarly, the present findings showed that all aforementioned zirconia crowns veneered with the three mentioned methods could withstand the maximum masticatory force [ 30 ], and all were clinically acceptable.

Although there was no statistically significant difference between the failure patterns of the three groups, the adhesive pattern was the most common pattern in the CAD-on group and the cohesive pattern was the most common in the layering and press-on groups. It was found that the layering and press-on methods established stronger interfacial bond between the zirconia coping and the ceramic veneer than CAD-on method. In agreement with this study, Kanat-Erturk *et al*. [ 24 ] observed that the cohesive failure pattern was more common with the press-on, and the adhesive failure pattern was more common with over-cemented file-spitting technique. Gostemeyer *et al*. [ 31 ] reported that the cohesive failure pattern occurred most frequently. Guess *et al*. [ 32 ] found that mixed cohesive and adhesive failure pattern occurred most frequently. However, the small sample size of the present study does not allow a comprehensive conclusion on the frequency of the failure patterns. 

The present study also evaluated surface roughness of crowns before and after aging. In this study, press-on group exhibited higher surface roughness before and after aging, followed by the CAD-on and the layering groups. In addition, the layering technique was performed manually by an experienced technician, which resulted in lower surface roughness, as it highly relies on the dental technician's skills. The higher surface roughness resulting from press-on could be due to the burning out of the wax, the removal of the investment material, the immersion in hydrofluoric acid solution, sandblasting, and the contouring sequences [ 25 ]. The results showed that aging did not significantly change the surface roughness in the three study groups. Tang *et al*. [ 7 ] investigated the effect of aging on the surface texture of veneering ceramics in zirconia copings. They observed changes in surface roughness and microscopic cracks after aging. Burgess *et al*. [ 33 ] investigated enamel wear opposing to zirconia crowns before and after aging. They noticed that aging did not significantly increase surface roughness.

Limitations of this study included the use of brass dies, which provided more mechanical support than natural teeth that did not simulate the oral cavity environment; therefore, studies using natural teeth are recommended. In addition, the present study did not evaluate fatigue, which should be considered in long-term studies in the oral cavity. Undoubtedly, artificial aging does not simulate the oral cavity environment identically in terms of time and temperature. Due to the *in vitro* nature of this study, the prosthesis was subjected to forces in only one direction (vertical), which does not simulate the complex forces in the oral cavity and therefore requires future in vivo studies. Storing the specimens in saliva might be of great help; therefore, further studies are recommended to use artificial saliva [ 17 ].

## Conclusion

Within the limitations of this *in vitro* study, it can be concluded that the fracture strength of zirconia crowns veneered through CAD-on, layering and press-on techniques was clinically acceptable. The layering technique creates zirconia crowns with the lowest surface roughness, followed by CAD-on and press-on methods. Nevertheless, surface roughness was clinically acceptable for all three methods. Aging has no significant effect on surface roughness in any of the three studied methods. Regarding the failure patterns, layering and press-on veneering methods showed more cohesive failure, while adhesive failure pastern was more frequent in CAD-on method. 

## References

[1] Gundugollu Y, Yalavarthy RS, Krishna MH, Kalluri S, Pydi SK, Tedlapu SK ( 2018). Comparison of the effect of monolithic and layered zirconia on natural teeth wear: An in vitro study. J Indian Prosthodont Soc.

[2] AlAmleh B, Lyons K, Swain M ( 2010). Clinical trials in zirconia: A systematic review. J Oral Rehabil.

[3] Sandhu R, Kheur M, Kheur S ( 2017). Effect of simulated chairside grinding procedures using commercially available abrasive agents on the surface properties of zirconia. J Indian Prosthodont Soc.

[4] Güngör MB, Nemli SK ( 2018). Fracture resistance of CAD/ CAM monolithic ceramic and veneered zirconia molar crowns after aging in a mastication simulator. J Prosthet Dent.

[5] Roumanas ED ( 2013). The clinical reliability of zirconia-based fixed dental prostheses appears acceptable but further research is necessary. J Evid Based Dent Prac.

[6] Nikzadjamnani S, Azari A, Niakan S, Namdar SF ( 2017). Fracture resistance of zirconia restorations with a modified framework design. J Dent (Tehran, Iran).

[7] Tang X, Tan Z, Nakamura T, Yatani H ( 2012). Effects of ageing on surface textures of veneering ceramics for zirconia frameworks. J Dent.

[8] White S, Miklus V, McLaren E, Lang L, Caputo A ( 2005). Flexural strength of a layered zirconia and porcelain dental all-ceramic system. J Prosthet Dent.

[9] Ishibe M, Raigrodski AJ, Flinn BD, Chung KH, Spiekerman C, Winter RR ( 2011). Shear bond strengths of pressed and layered veneering ceramics to high-noble alloy and zirconia cores. J Prosthet Dent.

[10] Torabi K, Vojdani M, Giti R, Taghva M, Pardis S ( 2015). The effect of various veneering techniques on the marginal fit of zirconia copings. J Adv Prosthodont.

[11] Beuer F, Schweiger J, Eichberger M, Kappert HF, Gernet W, Edelhoff D ( 2009). High-strength CAD/CAM-fabricated veneering material sintered to zirconia copings- a new fabrication mode for all-ceramic restorations. Dent Mater.

[12] Chaar M, Witkowski S, Strub J, Att W ( 2013). Effect of veneering technique on the fracture resistance of zirconia fixed dental prostheses. J Oral Rehabil.

[13] Kanat B, Çömlekoğlu EM, Dündar‐Çömlekoğlu M, Hakan Sen B, Özcan M, Ali Güngör M ( 2014). Effect of various veneering techniques on mechanical strength of computer controlled zirconia framework designs. J Prosthodont.

[14] Solá-Ruiz MF, Agustín-Panadero R, Campos-Estellés C, Labaig-Rueda C ( 2015). Post-fatigue fracture resistance of metal core crowns: Press-on metal ceramic versus a conventional veneering system. J Clin Exp Dent.

[15] Gaonkar SH, Aras MA, Chitre V ( 2020). An in vitro study to compare the surface roughness of glazed and chairside polished dental monolithic zirconia using two polishing systems. J Indian Prosthodont Soc.

[16] Amer R, Kürklü D, Johnston W ( 2015). Effect of simulated mastication on the surface roughness of three ceramic systems. J Prosthet Dent.

[17] Hussein AI, Hajwal SI, Ab-Ghani Z ( 2016). Bacterial adhesion on zirconia, lithium desilicated and gold crowns in vivo study. Adv Dent Oral Health.

[18] Snyder MD, Hogg KD ( 2005). Load-to-fracture value of different all-ceramic crown systems. J Contemp Dent Pract.

[19] Kobayashi K, Kuwajima H, Masaki T ( 1981). Phase change and mechanical properties of zro2-y2o3 solid electrolyte after ageing. Solid State Ionics.

[20] Rupawala A, Musani SI, Madanshetty P, Dugal R, Shah UD, Sheth EJ ( 2017). A study on the wear of enamel caused by monolithic zirconia and the subsequent phase transformation compared to two other ceramic systems. J Indian Prosthodont Soc.

[21] Lughi V, Sergo V ( 2015). Low temperature degradation -aging- of zirconia: A critical review of the relevant aspects in dentistry. Dent Mater.

[22] Mitov G, Anastassova-Yoshida Y, Nothdurft FP, Von See C, Pospiech P ( 2016). Influence of the preparation design and artificial aging on the fracture resistance of monolithic zirconia crowns. J Adv Prosthodont.

[23] Mohaghegh M, Firouzmandi M, Ansarifard E, Ramazani L ( 2020). Marginal fit of full contour zirconia in different thicknesses and layered zirconia crowns. J Int Soc Prevent Communit Dent.

[24] Kanat-Erturk B, Comlekoglu EM, Dundar-Comlekoglu M, Ozcan M, Gungor MA ( 2015). Effect of veneering methods on zirconia framework-veneer ceramic adhesion and fracture resistance of single crowns. J Prosthodont.

[25] Kumchai H, Juntavee P, Sun AF, Nathanson D ( 2021). Effect of veneering ceramic and methods on failure load of veneered zirconia. Appl Sci.

[26] Hung C, Huang Y ( 2012). Effect of veneering techniques on ceramic fracture of zirconia restoration. J Prosthodont Imp.

[27] Pandurangan KK, Veeraiyan DN, Nesappan T ( 2020). In vitro evaluation of fracture resistance and cyclic fatigue resistance of computer-aided designon and hand-layered zirconia crowns following cementation on epoxy dies. J Indian Prosthodont Soc.

[28] Ezzat YE, Al-Rafee MA ( 2020). Effect of veneering material and technique on the fracture resistance of porcelain-veneered zirconia crowns. Saudi J Oral Sci.

[29] Sim J, Lee W, Kim J, Kim H, Kim W ( 2016). Evaluation of shear bond strength of veneering ceramics and zirconia fabricated by the digital veneering method. J Prosthodont Res.

[30] Drummond JL (2003). Ceramic behavior under different environmental and loading conditions. Dental Materials in vivo: Aging and Related Phenomena.

[31] Gostemeyer G, Jendras M, Borchers L, Bach F, Stiesch M, Kohorst P ( 2012). Effect of thermal expansion mismatch on the ytzp/veneer interfacial adhesion determined by strain energy release rate. J Prosthodont Res.

[32] Guess P, Kulis A, Witkowski S, Wolkewitz M, Zhang Y, Strub J ( 2008). Shear bond strength between different zirconia cores and veneering ceramics and their susceptibility to thermocycling. Dent Mater.

[33] Burgess J, Janyavula S, Lawson N, Lucas T, Cakir D ( 2014). Enamel wear opposing polished and aged zirconia. Oper Dent.

